# Prey capture by the non‐native carnivorous pitcher plant *Sarracenia purpurea* across sites in Britain and Ireland

**DOI:** 10.1002/ece3.9588

**Published:** 2022-12-13

**Authors:** Rebecca Whatmore, Paul J. Wood, Ciara Dwyer, Jonathan Millett

**Affiliations:** ^1^ Geography and Environment Loughborough University Loughborough UK; ^2^ Centre for Environmental and Climate Science Lund University Lund Sweden

**Keywords:** carnivorous plants, invasive alien species (IAS), non‐native species, prey capture, *Sarracenia purpurea*

## Abstract

The carnivorous pitcher plant *Sarracenia purpurea* is native to North America, but has been introduced into Europe, where it is now widespread. Understanding of how this species functions in its non‐native range is limited. We measured pitcher morphology and prey capture by *S. purpurea* in its non‐native range in Britain and Ireland. Pitchers were removed from different plants at each of six bogs covering the species range in Britain and Ireland (*n* = 10 pitchers per site). For each pitcher we counted and identified every prey item and took measurements of morphology. We also compiled prey capture data for existing studies in Europe and North America. Prey capture characteristics varied between sites in Britain and Ireland. The amount of prey captured varied 20‐fold between sites and was partially explained by differences in pitcher size; larger pitchers caught more prey. The primary prey was Formicidae, Diptera and Coleoptera. At the rank of order, prey composition varied between bogs, some contained mainly Formicidae, some mainly Diptera and some a mix. Prey capture was less evenly distributed at some bogs compared to others, suggesting more specialization. There was no overall difference in prey capture (composition or evenness) at the rank of order between plants in Europe compared to those in North America. At the rank of species, prey capture varied between populations even within the same order. This study demonstrates a large amount of variability between sites in prey capture characteristics. This may reflect different site characteristics and/or plant strategies, which will likely impact plant function, and may impact the inquiline community. In terms of prey capture at the rank of order, *S. purpurea* functions identically in its non‐native range. This supports its use as a model system in a natural experiment for understanding food webs.

## Graphical abstract

We surveyed prey capture by the carnivorous pitcher plant *Sarracenia purpurea* at six sites in its non‐native range. We demonstrated that prey capture varies in ecologically significant ways between populations, but overall there is no difference in prey capture patterns in the plants non‐native compared to native range.

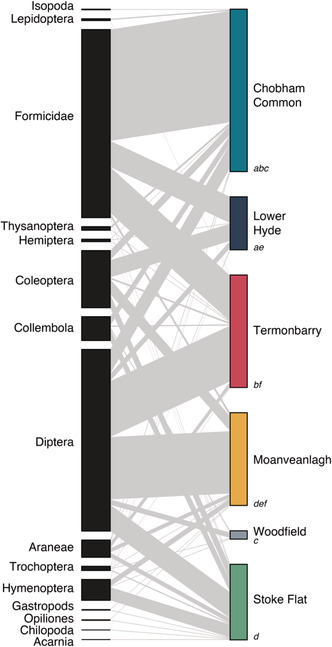

## 1 INTRODUCTION

Human movement of plants around the Earth through global trade networks and colonialism has resulted in the homogenization of flora (Chapman et al., 2017). When plant species establish in areas outside of their native area, they can have important negative impacts (Vilà et al., 2011). Invasive non‐native species are, as such, considered one of the biggest threats to global biodiversity (Pyšek et al., 2020). After they arrive in a new location, non‐native plants interact with extant flora and fauna (Van Kleunen et al., 2018). These biotic interactions determine non‐native plant fitness, and so are key to their continued survival and fitness. This can be important where interactions differ in the introduced compared to the native range (Van Kleunen et al., 2018). For example, changes in pollinator, herbivore and plant–plant interactions in the introduced range can expand the realized niche of non‐native species (Mitchell et al., 2006). Understanding of these trophic interactions is, however, poorly understood despite this being a key impact of non‐native plant species (Vilà et al., 2019). Such differences in ecology in the native vs non‐native range can, in addition, provide more general insights into plant ecological function, because plant introductions represent an unplanned natural experiment.

The carnivorous pitcher plant *Sarracenia purpurea* L. provides a particularly compelling study system for understanding changes in trophic interactions in a plant's native vs non‐native range. *Sarracenia purpurea* is a herbaceous, perennial, carnivorous pitcher plant native to bogs and nutrient‐poor fens in North America (Wherry, 1929). The leaves are formed into lidless cup‐shaped pitchers, which produce nectar from extrafloral nectaries (Vogel, 1997) to which insect prey are attracted (Bennett & Ellison, 2009). Captured invertebrate prey fall into rainwater held in the pitcher and decompose (Slack, 1979). The pitchers host inquiline detritivore communities that facilitate prey digestion, the nutrients from which the plant uses for growth and reproduction (Chapin & Pastor, 1995). These inquiline communities are model systems for understanding food‐web dynamics (Baiser et al., 2013; Gray et al., 2012; Sirota et al., 2013; Srivastava et al., 2004).

*Sarracenia purpurea* was introduced into Europe in the late 19th century (Foss & O'Connell, 1985) and is now present on >100 sites in Europe, including 38 in Britain and Ireland (Walker, 2014); as far as it is known, introduction has always been deliberate. On some sites *S. purpurea* can be invasive, attaining populations of >100,000 plants (Adlassnig et al., 2010), where local impacts on native flora can be severe (Sanderson, 2012). *Sarracenia purpurea* is not known to have ever moved from one site to another through natural dispersal (Long, 2013), so it can be considered a naturalized species (sensu Blackburn et al., 2011). The ecology of *S. purpurea* in its non‐native range is of interest for two reasons: its invasiveness on protected sites is of nature conservation importance (Walker, 2015), and differences in ecology in its native vs non‐native range presents an interesting natural experiment for understanding food‐web function. However, knowledge of *S. purpurea* diet, even in the species' native range, is limited. This limits the study of other aspects of ecology because prey capture and diet are fundamental to *S. purpurea* ecology.

Surprisingly, few studies exist of prey capture by *S. purpurea* in its native range, and even fewer in its non‐native range. Ellison and Gotelli (2009) demonstrated that the diet of *Sarracenia* sp. is relatively narrow and consists mainly of species in the family Formicidae (Order: Hymenoptera) with some Diptera and Coleoptera. This synthesis included only six studies of *S. purpurea* prey capture, demonstrating the paucity of such studies. In European populations prey capture has been observed in a number of studies (e.g. Franklin et al., 2017; Owen & Taylor, 1994), but as far as we are aware it has only been quantified in a single study to‐date—Zander (2017). This makes evaluation of prey capture in Europe, and comparison in the species native and non‐native range impossible. Pitcher morphology is very variable, much of which is explained by differences in climate, and also related to prey capture. In their native range, *S. purpurea* pitchers are larger on wetter sites, tall and thin on warmer sites and short with big mouths on cooler sites (Ellison et al., 2004). Cresswell (1993) found that at a single site, larger pitchers caught more prey. It would be expected that variation in pitcher morphology between sites would also result in differences in prey capture, but the lack of comparative studies means that this is not yet confirmed. The lack of such fundamental understanding of the species ecology in its introduced range undermines the use of this system as a model for understanding inquiline dynamics.

In this study we quantified prey capture by *S. purpurea* growing in naturalized populations across Britain and Ireland to address the following four questions:

1. What prey taxa does *S. purpurea* catch in Britain and Ireland?
2. Does *S. Purpurea* diet, measured as the amount (number of prey items), breadth (taxonomic richness), composition, and specialization (evenness) of captured prey vary between sites?
3. Does pitcher morphology affect the amount of prey captured?
4. Does the composition of captured prey differ in the species native and non‐native range?

## 2 METHODS

**FIGURE 1:**
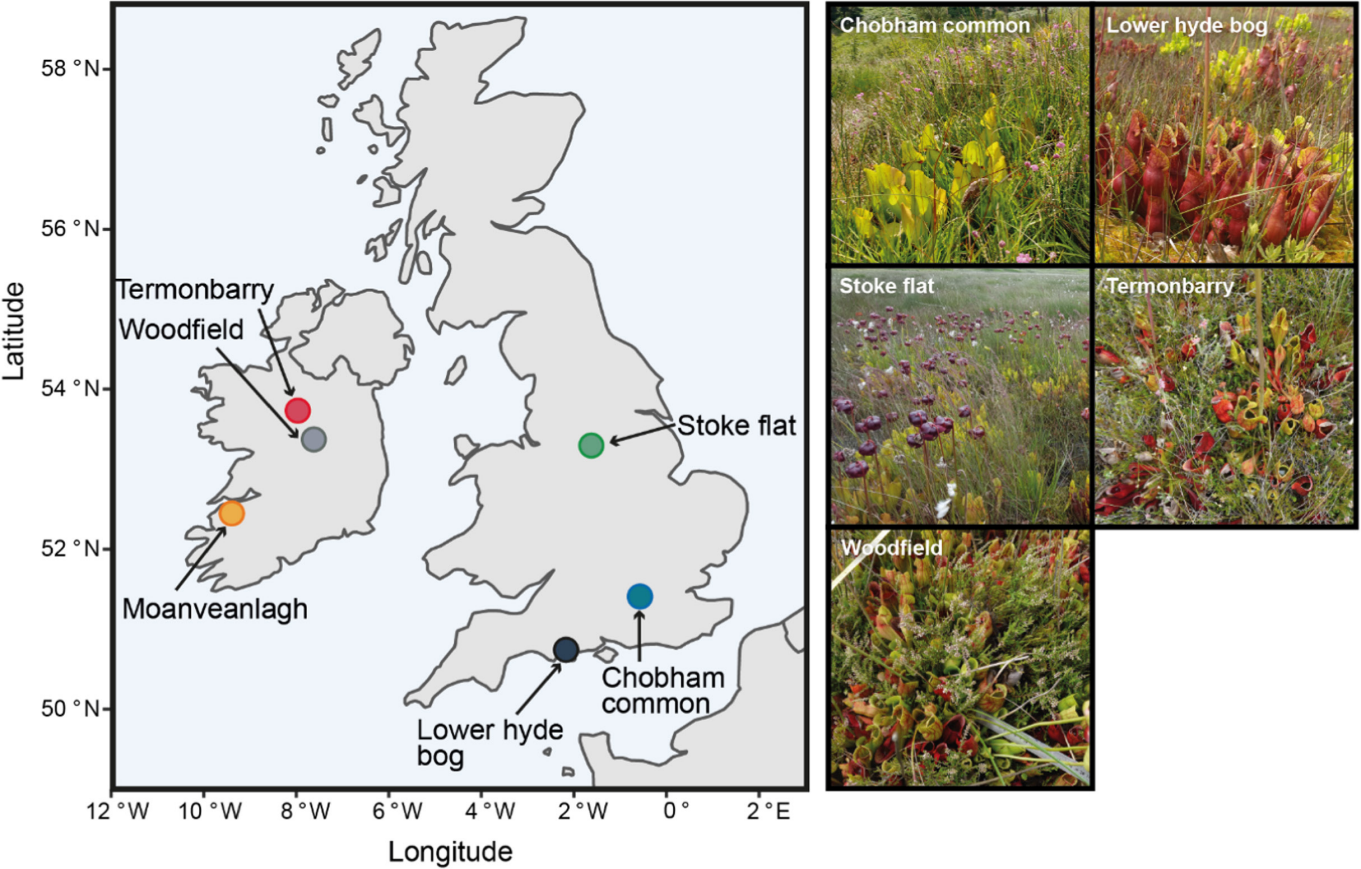
Locations and indicative photos of study sites.

Between July 13 and August 30, 2016, six *S. purpurea* populations were sampled across Britain and Ireland (Figure 1); these sites were chosen to cover a broad geographic range, to encompass likely variability in populations. We tried to keep sampling as close in time as possible, so that leaves will be of similar ages, with similar amounts of time available for prey capture. Five of the sites were sampled within a two‐week period. One site, Stoke Flat, had to be sampled later due to logistical considerations. Results from this site should be interpreted in this context. Details of each site are as follows.

**Chobham Common (Britain, sampled July 13)** is situated at an altitude of 39 m a.s.l and consists of 585 hectares of temperate lowland heath. Chobham Common is an SSSI and the largest National Nature Reserve in Surrey, England. The reserve supports a longstanding population of approximately 20 healthy, adult *S. purpurea* plants clustered on one large hummock. A large patch of juveniles is also present despite active attempts to halt the spread through the deflowering of adults.

**Stoke Flat (Britain, sampled August 30)** is situated at an altitude of 275 m a.s.l, above the Froggatt Edge escarpment in the Peak District in England, and is a small seepage mire at the edge of a beech forest. This site supported a population of approximately 100 plus *S. purpurea* individuals. These have since been removed through treatment with herbicide.

**Lower Hyde (Britain, sampled July 21)** is a lowland valley mire located in Wareham Forest, Dorset, England at an altitude of 34 m a.s.l. This population, previously contained approximately 500 plus pitchers, derived from seven individual plants that were introduced in the 1980s with seeds also being planted in the 1990s (Bailey & McPherson, 2016). Most of the plants have now been removed using hand‐pulling, but a small area (<100 m^2^) has been left for research purposes.

**Moanveanlagh (Ireland, sampled July 29)** is an active raised bog in Ireland at an altitude of 12 m a.s.l. The western edges of the bog have experienced substantial drainage due to peat cutting. Despite this, Moanveanlagh supports a large *S. purpurea* of approximately 10,000 individuals or more.

**Termonbarry (Ireland, sampled July 28)** is a raised bog in Ireland which has supported *S. purpurea* population since 1906 when seed was introduced. From these individuals, multiple populations were established at other Irish bogs in the 1960s (Taggart et al., 1990). This site has been subjected to large‐scale industrial peat cutting that has reduced the expanse of the pitcher population from 35 to 10 ha. This area is densely populated, however with an estimate of 100,000 or more individuals being deemed conservative.

**Woodfield (Ireland, sampled July 26)** is a raised bog in Ireland that supports one of the largest populations in Britain and Ireland. Approximately 230,000 plus individuals have become firmly established despite threats from extensive peat cutting operations (Duignan, 2013). This population was introduced from three individuals transplanted from Coolatore bog. The site also supports the only known wild‐growing population of California Pitcher (*Darlingtonia californica)* in Britain and Ireland.

For each population, a pitcher was sampled from each of 10 plants, chosen at equal intervals along a transect of varied length depending on the extent of *S. purpurea* cover. For sites in England the transect was 30 m, in Ireland transects were 100 m. We tried to standardize pitcher selection as much as possible, mainly by avoiding those plants which were produced the previous year. However, it is recognized some may be more mature than others, which will give more time for prey capture (but also for decomposition). We would expect this impact to be even across a population, but it may be important when considering data from Stoke Flat, which was sampled 1 month after the other sites. For each plant, the maximum diameter of each rosette was recorded along the longest axis and vascular vegetation cover was estimated in 1 m^2^ surrounding each rosette. A current‐year, fully developed pitcher was then separated from the base of the rosette by gently pulling them from the base of the stem by hand to remove the full length of the pitcher. Excess pitcher solution was carefully pipetted out, ensuring as best as possible that no invertebrates were removed in the process. Each pitcher was then stored in a sealed ziploc bag. Pitchers were transported to an indoor location for measurement. Invertebrate prey were flushed out and preserved in ethanol. All invertebrates present within each pitcher were counted and identified to the finest possible taxonomic resolution.

To complement our prey capture data, and to provide understanding of the differences between the populations we made measurements of the vegetation in which each *S. purpurea* plant was growing and of characteristics of each *S. purpurea* plant. We estimated canopy cover visually, and measured canopy light interception by comparing the intensity of photosynthetically active radiation (Skye Instruments Quantum PAR sensor = 400–700 nm) above the plant canopy and at ground surface underneath the plant canopy (*n* = 5 locations per pant). We measured pitcher water pH using pH test strips. Pitcher chlorophyll content was measured using optical absorbance (620 and 940 nm wavelength) with a handheld meter (Hansatech CL‐01 chlorophyll content meter) which gives a measure of relative chlorophyll content. The specific leaf area of each pitcher was measured by removing nine 5 mm diameter disks from each pitcher using a biopsy punch (three each from the keel, top of the pitcher and bottom of the pitcher). These were subsequently dried at 60°C and weighed. We characterized the morphology of the pitchers by measuring: pitcher length, diameter of the pitcher mouth, thickness of the pitcher lip, maximum width of the pitcher and the width of the keel. These are the same measures used by Ellison and Gotelli (2002).

### 2.1 Data analysis

We addressed the following four data analysis questions: (1) does prey capture (amount, breadth and composition) vary between populations in Britain and Ireland, (2) does the degree of prey specialization vary between populations in Britain and Ireland, (3) can differences in prey capture be explained by variability in pitcher morphology, and (4) does prey composition vary in the non‐native range compared to the native range (using analysis of secondary data). Data analysis was undertaken using R version 4.2.1 (R Core Team, 2022). We undertook analysis using taxonomic data at the order level, because this was the finest resolution to which we were able to identify all prey items. This also matches approaches in previous *S. purpurea* prey capture studies. Like previous studies we separated Hymenoptera into Formicidae and non‐Formicidae Hymenoptera because Formicidae are a particularly important component of *Sarracenia* prey.

To address question one, we first compared the amount of prey captured between sites using Analysis of Variance (ANOVA) followed by Fisher's LSD (Least Significant Difference) post‐hoc comparisons. For the ANOVA we used the function ‘aov’ in the base package ‘stats’ (R Core Team, 2022), and for the LSD post‐hoc comparisons we used the function ‘LSD.test’ in the package ‘agricolae’ (de Mendiburu, 2021). To determine, how diet breadth varies, we compared the richness of prey taxa (order level plus Formicidae) captured between sites using ANOVA and Fishers LSD. We compared prey community composition (relative frequency of prey types) between sites using permutational MANOVA (PERMANOVA). PERMANOVA uses permutation tests to determine whether the observed dissimilarities in prey composition between different sites are greater than that would be expected by chance. We used the function ‘adonis2’ in the package ‘vegan’ (Oksanen et al., 2022). We chose the Morisita–Horn index to generate these dissimilarity matrices of the prey community composition, as this method is robust to unequal sample sizes (Barwell et al., 2015; Krebs, 1999).

To address question two we used the evenness of prey composition to evaluate the potential for specialization of prey capture using Pielou's evenness. We used the function ‘diversity’ in the package ‘vegan’ (Oksanen et al., 2022). A value of 1 indicates that the pitchers contained prey from a single taxonomic group, suggesting a high degree of specialization; a value of 0 indicates that the prey are equally distributed between taxonomic groups, suggesting that the plant is more generalist. Ellison and Gotelli (2009) used a similar measure of evenness to establish the potential for prey specialization between carnivorous plant species. It is important to note that the evenness of prey capture does not in itself confirm specialization (or lack of) without understanding the availability of prey in the local environment (Ellison & Gotelli, 2009). However, how to best measure specialization in carnivorous plants is still not yet clearly determined (Darnowski et al., 2017). Prey evenness is a useful measure in the absence of data on available prey (Ellison & Gotelli, 2009). Differences in Pielou's evenness between sites were tested using ANOVA (function ‘aov’ in the base package ‘stats’ (R Core Team, 2022)), followed by Fisher's LSD (function ‘LSD.test’ in the package ‘agricolae’ (de Mendiburu, 2021)).

Vegetation and plant characteristics were compared between sites using analysis of variance (ANOVA), followed by Fisher's LSD post‐hoc tests. For the ANOVA we used the function ‘aov’ in the base package ‘stats’ (R Core Team, 2022), and for the LSD post‐hoc comparisons we used the function ‘LSD.test’ in the package ‘agricolae’ (de Mendiburu, 2021). To answer the question three, we used multiple regression with number of prey items as the dependant variable, and pitcher lip with, mouth width, keel width, total width, length and specific leaf area (SLA) as the independent variables. Test assumptions were checked using diagnostic plots. To determine whether differences in the amount of prey captured could instead be a function of *S. purpurea* population size we used Spearman's rank correlation coefficient (calculated by hand), comparing rank estimated population size and rank prey abundance.

To determine whether prey capture differed in Europe compared to North America, addressing question four, we synthesized existing data for prey capture by *S. purpurea* plants in North America and Europe. This included the studies originally synthesized by Ellison and Gotelli (2009) and additional studies obtained through a systematic search of the literature. This search was undertaken in Google, Google Scholar and Web of Science using the search terms “*Sarracenia purpurea*” and “prey”, and searches of the references of relevant papers. A total of seven previous studies were found, covering nine locations: Bennett and Ellison (2009), Cresswell (1993), Folkerts (1992), Heard (1998), Judd (1959), Newell and Nastase (1998), Zander (2017). Data on prey capture were extracted and grouped into the taxonomic classes used in the present study. We pooled all data from a single site to create one record of prey capture composition per site. Differences in prey composition between sites in North America and those in Europe were compared using PERMANOVA and analysis of differences in the evenness of prey capture between the two continents by calculating Pielou's evenness followed by ANOVA. For the PERMANOVA, we used the function ‘adonis2’ in the package ‘vegan’ (Oksanen et al., 2022), and for Pielou's evenness we used the function ‘diversity’ in the package ‘vegan’ (Oksanen et al., 2022).

## 3 RESULTS

We counted a total of 848 prey items across all 59 *S. purpurea* pitchers. We were able to identify half (419 individuals) to species. This taxonomic resolution varied depending on the taxonomic grouping. We were able to identify the majority of coleoptera (83 out of 96 individuals), non‐Formicidae Hymenoptera (25 out of 35 individuals) and Formicidae (300 out of 316 individuals) to species but were only able to identify 5 out of 305 Diptera to species due to the extent of decomposition. Of the seven most abundant species, none were present in pitchers at every site, four were present at a single site and one species was present at only two sites (Table 1). At four sites only one of these species was found in *S. purpurea* pitchers. None of these species were found at Woodfield.

**TABLE 1:** Most abundant prey species identified from *Sarracenia purpurea* pitchers located at six sites in Britain and Ireland (site details in Figure 1).

Order	Family	Genus	Species	Total	MV	LH	CC	TB	WF	FR
Coleoptera	Carabidae	*Ocys*	*harpaloides*	17	0	0	0	0	0	17
Coleoptera	Chrysomelidae	*Sphaeroderma*	*testaceum*	36	0	27	8	1	0	0
Coleoptera	Scraptiidae	*Anaspis*	*maculata*	11	11	0	0	0	0	0
Hymenoptera	Formicidae	*Formica*	*picea*	25	0	25	0	0	0	0
Hymenoptera	Formicidae	*Lasius*	*psammophilus*	164	0	0	164	0	0	0
Hymenoptera	Formicidae	*Myrmica*	*rubra*	81	0	0	5	76	0	0
Hymenoptera	Formicidae	*Myrmica*	*scabrinodis*	29	5	19	3	0	0	2

*Note*: Data presented are total number of prey species recorded for each site, and overall, for the most abundant (*n* > 10 in total) individuals which could be identified to species level. Site codes: MV, Moanveanlagh; LH, lower Hyde bog; CC, Chobham common; TB, Termonbarry; WF, Woodfield; FR, stoke Flat.

**FIGURE 2:**
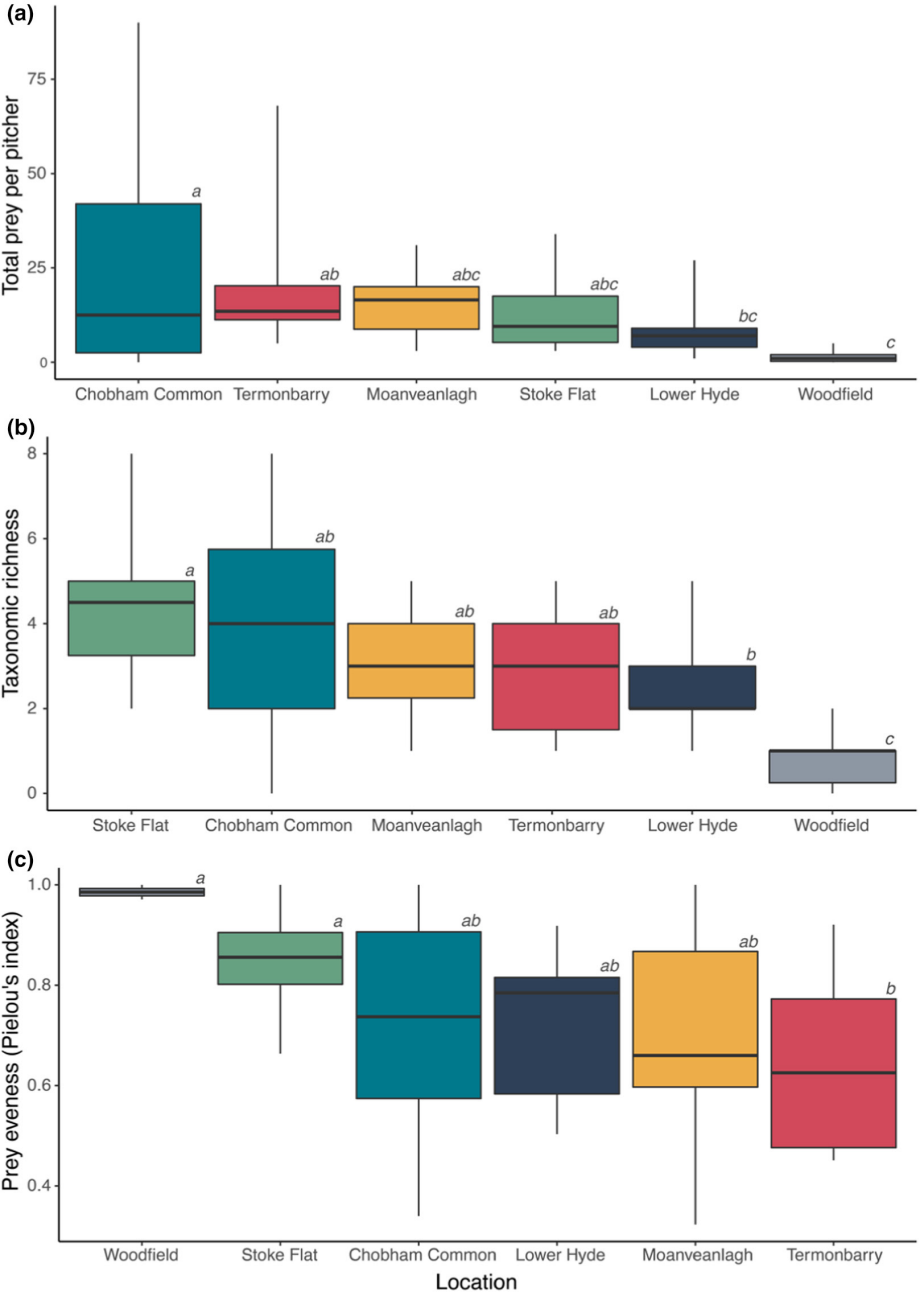
Prey capture characteristics of *Sarracenia purpurea* pitchers growing on six sites in Britain and Ireland. Data presented are: (a) total number of invertebrates found in each pitcher, (b) number of different taxonomic groups in each pitcher, and (c) Pielou's evenness of invertebrate groups, where a value of 1 indicates that the pitchers contained prey from a single taxonomic group and a value of 0 indicates that the prey are equally distributed between taxonomic groups. For sites with different letters the differences in means are statistically significant (Fisher's LSD: *p* < .05). Boxplots show 5th, 25th, 50th, 75th and 95th percentile for each site.

The amount of prey captured varied between sites. The total number of prey items found in *S. purpurea* pitchers varied almost 20‐fold, differences which were statistically significant (Figure 2a, ANOVA: *F*_5,53_ = 2.755, *p* = .028). Plants at Woodfield contained the fewest prey items; those at Chobham Common contained most. At the Order level pitchers at Stoke Flat contained the highest number of taxa on average and Lower Hyde Bog the lowest (Figure 2b). This pattern was the same at the species level. There was no correlation between *S. purpurea* population size and the number of prey captured (Spearman's rank correlation: rho = −0.49, df = 4, *p* = .32).

**FIGURE 3:**
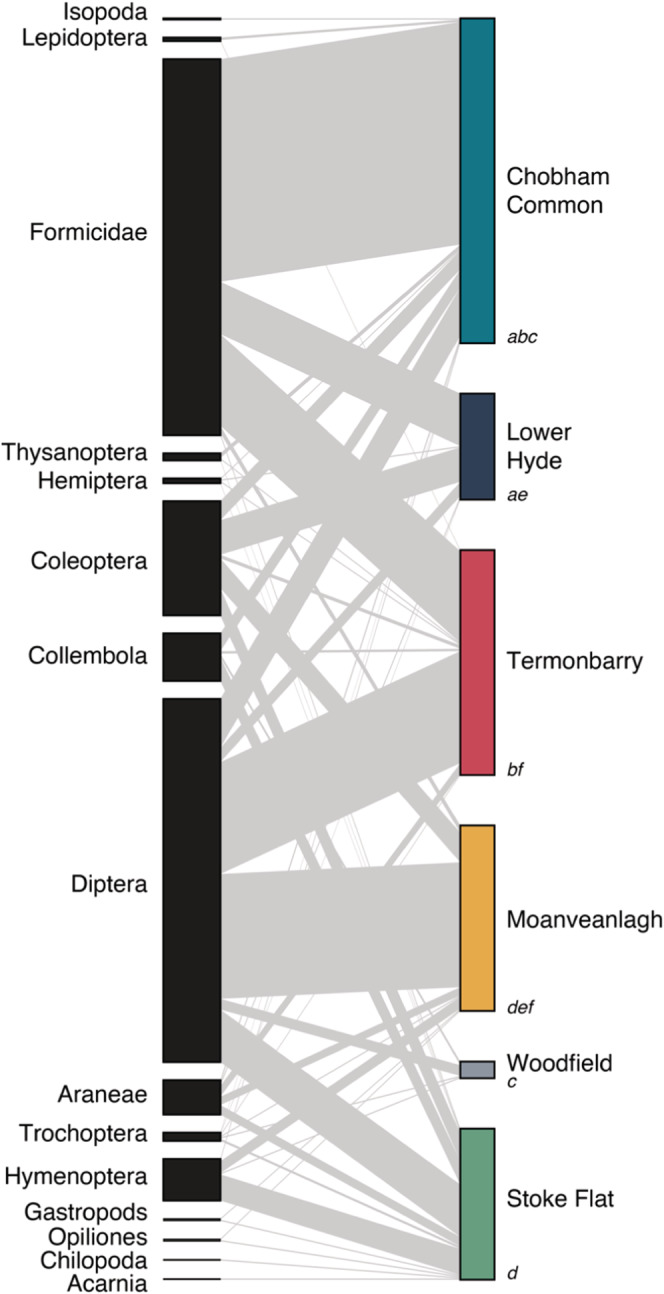
Bipartite food webs showing the observed plant–prey interactions in terms of the relative frequency of prey captured by *Sarracenia purpurea* growing at different sites (see Figure 1 for locations). The right‐hand side contains all the locations (*n* = 6). The length of the rectangle is relative to average total number of prey items per pitcher. The left‐hand side shows all prey types (*n* = 15). The length of the rectangle is relative to the number of prey in that group across all sites. Gray lines connecting populations to prey types show predatory interactions, with the width of the line representing the proportion of total capture for each population corresponding to a given prey type. For sites with different letters the prey community composition is significantly different (pairwise Adonis, *p* < .05).

As the taxonomic resolution varied between taxonomic group, we assessed variation in the breadth of plant diet at the Order level (except for Formicidae). At this level diet breadth was different between sites. Pitchers at Woodfield contained prey in, on average, only 1 group, but it is difficult to determine whether this was a consequence of low prey capture. Pitchers at Lower Hyde contained prey from, on average, 2.4 groups, whereas those from Stoke Flat contained prey from, on average, 3.8 groups. The differences in number of taxa caught were statistically significant (Figure 2b, ANOVA: *F*_5,53_ = 5.675, *p* = .001). Pitcher contents were primarily composed of Formicidae and Diptera, in relatively equal proportions overall (Figure 3); Coleoptera were a significant component of pitcher's prey at some sites. The composition of the prey in pitchers was different at different sites (Figure 3, PERMANOVA: F_5,49_ = 4.96, *p* = .001). At some, prey capture was dominated by a single taxonomic group—Formicidae at Chobham Common and Diptera at Moanaveagh. At others, prey capture was split primarily between two groups—Formicidae and Diptera at Termonbarry, Formicidae and Coleoptera at Lower Hyde. At Stoke Flat, prey capture was more evenly distributed among five groups.

There is some evidence that the extent of prey specialization (measured with evenness of prey capture) also varied between sites, but this is more equivocal. The differences between evenness of prey capture were quite large, varying from 0.98 at Woodfield, 0.85 at Stoke Flat and 0.64 at Termonbarry (Figure 2c), but these differences were not statistically significant (ANOVA: *F*_5,37_ = 1.956, *p* = .108). At the Species level, the identity of the most abundant species was different at each site (Table 1).

Our systematic literature search identified prey composition data from a total of 16 sites (9 in North America and 7 Europe including 6 from the present study). We found no evidence that pitcher prey contents differed between plants growing in North America compared to those growing in Europe at the order level (PERMANOVA: *F*_1,12_ = 1.7284, *p* = .12). In addition, there was no difference in the evenness of pitcher prey contents in *S. purpurea* growing in North America compared to *S. purpurea* growing in Europe. Mean Pileou's evenness index for pitchers in North America was 0.61 and for those in Europe was 0.60 (*t*‐test: *t*_12_ = 0.337, *p* = .74).

There was little difference between sites in vegetation cover and light interception (Table 2). Despite these similarities, plants varied approximately twofold in size (total plant diameter). Pitchers also varied in size between sites. Those at Chobham Common were the largest, being longer and having wider mouthed pitchers and keels than the other sites. The pitchers of plants at Woodfield were the smallest, being approximately half the size of those at Chobham Common for the same measures. Pitcher fluid pH was generally similar between sites except for Stoke Flat where pH was significantly higher. Chlorophyll content was higher for pitchers at Chobham Common and Stoke Flat. There were no statistically significant differences in pitcher SLA between sites. Pitcher morphology influenced the amount of captured prey. The number of prey items in a pitcher was the highest for pitchers with wider mouths and lower SLA (Table 3).

**TABLE 2:** Vegetation and *Sarracenia purpurea* characteristics for each of six study sites.

Site	Veg. Cover (%)	Light intercept. (%)	Plant dia. (cm)	pH	Chlorophyll content	SLA (cm^−1^ g^−1^)	Pitcher morphology				
							Lip width (mm)	Mouth width (mm)	Keel width (mm)	Total width (mm)	Length (mm)
England											
Chobham Common	27.50	39 ± 12	37.85 ± 6.45^ab^	5.55 ± 0.09^b^	10.25 ± 3.57^b^	141 ± 17	2.74 ± 0.25^b^	29.44 ± 2.35^a^	23.19 ± 2.48^a^	48.41 ± 3.12^a^	277.50 ± 18.37^a^
Stoke Flat	22 ± 4	35 ± 5	46.50 ± 5.68^a^	5.20 ± 0.13^b^	27.29 ± 1.35^a^	173 ± 10	1.84 ± 0.16^c^	21.34 ± 1.45^bc^	13.49 ± 1.56^b^	41.92 ± 3.09^ab^	220.50 ± 7.13^b^
Lower Hyde	35 ± 11	19 ± 5	27.17 ± 3.55^bc^	6.33 ± 0.17^a^	2.72 ± 0.39^c^	166 ± 3	3.44 ± 0.31^a^	20.74 ± 0.99^bc^	17.11 ± 1.56^b^	41.17 ± 2.17^b^	182.22 ± 6.19^c^
Ireland											
Moanveanlagh	40 ± 7	33 ± 7	19.20 ± 1.12^c^	5.70 ± 0.35^b^	3.92 ± 0.54^c^	137 ± 6	2.52 ± 0.15^b^	24.70 ± 0.96^b^	14.97 ± 0.91^b^	37.55 ± 1.81^b^	165.50 ± 7.97^cd^
Termonbarry	46 ± 4	15 ± 3	27.60 ± 2.88^bc^	5.40 ± 0.12^b^	2.89 ± 0.24^c^	150 ± 4	2.63 ± 0.27^b^	22.52 ± 1.10^b^	14.97 ± 1.64^b^	38.81 ± 2.44^b^	177.00 ± 7.93^c^
Woodfield	34 ± 5	20 ± 4	26.35 ± 4.52^bc^	5.70 ± 0.20^b^	2.24 ± 0.43^c^	152 ± 6	2.57 ± 0.17^b^	17.50 ± 0.95^c^	7.88 ± 0.53^c^	29.76 ± 1.67^c^	139.50 ± 10.07^d^
ANOVA results											
*F*_5,53_	2.15	2.33	4.89	3.63	36.98	2.31	5.09	8.41	10.13	6.28	21.62
*p*	.07	.06	.001	.007	<.001	.06	.001	<.001	<.001	<.001	<.001

*Note*: Presented are the cover and percentage of incident light intercepted by the surrounding plant canopy, the diameter of individual *S. purpurea* plants, pitcher fluid pH, pitcher chlorophyll content, pitcher specific leaf area (SLA) and measures of pitcher morphology. Results of analysis of variance (ANOVA) tests for differences between sites are presented. Statistically significant differences between sites in each measure (Fisher's least significant difference) are indicated by different superscript letters.

**TABLE 3:** Results of multiple regression analysis for relationship between pitcher morphology and prey capture.

	Estimate	Standard error	*t*‐value	*p*
Intercept	−0.363	0.506	−0.718	.476
Lip width (mm)	−0.083	0.077	−1.079	.286
Mouth width (mm)	0.049	0.018	2.644	**.011**
Keel width (mm)	0.029	0.017	1.713	.093
Total width (mm)	−0.015	0.014	−1.101	.276
Pitcher length (mm)	−0.002	0.002	−0.894	.376
Specific Leaf Area (g cm^2^)	0.006	0.002	2.458	**.017**

*Note*: For the overall model *F*_6,52_ = 2.447, *p* = .037, adjusted *R* ^2^ = 0.13. Significant (*p* < .05) parameters are emboldened.

## 4 DISCUSSION

We quantified prey capture by *S. purpurea* using analysis of pitcher contents of plants growing at six sites in the species' non‐native range. Surprisingly, no study has previously compared *S. purpurea* prey capture between sites. We found clear evidence that prey capture by *S. purpurea* growing in its non‐native range varied between populations, in terms of amount, composition and breadth. We found limited evidence of differences in specialization. Overall, prey were primarily insects in the groups Formicidae, Diptera and to a lesser extent Coleoptera, which is in good agreement with previous studies (Ellison & Gotelli, 2009). *Sarracenia purpurea* in Britain and Ireland appear to be on average slightly specialist in their prey capture, which is in good agreement with Ellison and Gotelli's (2009) assessment of *Sarracenia* sp. The contribution of each taxonomic group varied between populations. At some populations prey capture was dominated by either Formicidae or Diptera, at others the diet was more varied. Our data also enable quantitative comparison of prey capture between continents in the species' native and non‐native range. We found no discernible difference overall in prey capture characteristics (at the rank of Order) in the non‐native range of *S. purpurea*, in comparison to prey capture in the plant's native range. The unprecedented taxonomic detail of our study, however, demonstrates high variation in diets between populations at finer (i.e. species) taxonomic resolutions, that is not captured by comparison at coarser resolutions (i.e. order). Thus, diets will very likely vary between continents in respect to species composition.

Carnivorous plant prey capture and nutrient uptake can vary between species (Ellison et al., 2003; Karlsson et al., 1994), populations (Millett et al., 2015) and individuals (Krowiak et al., 2017), due to factors such as the degree of investment in prey capture (Zamora, 1995), nutrient status of habitats (Millett et al., 2012) and the availability of prey (Krowiak et al., 2017). We show that for *S. purpurea* growing in Britain and Ireland the amount of prey captured varies greatly between populations on different sites. Prey composition also varies, which might impact nutrient uptake due to differences in nutritional value and digestibility of different insect species (Pavlovič et al., 2016). These differences might be important for plant function because prey capture provides a significant component of *S. purpurea* nutrition (Ellison & Gotelli, 2001). We did not measure background prey availability at each site, so cannot evaluate the extent that differences are because of different prey populations at each site, or differences in prey selection or attraction by *S. purpurea* at different sites. Some of this difference is, however, explained by differences in size, the smallest plants (at Woodfield) caught the least prey, the largest plants (at Chobham Common) caught the most prey. This is similar to findings by Cresswell (1993) who found that, at a single site in the plant's native range, larger pitchers caught more prey and a greater mass of prey. For passive traps, such as pitchers, a larger trap size is clearly likely to result in higher capture rates, but this might not be the only controlling factor. For example, larger pitchers might also produce more nectar, which is used as a prey attractant in *S. purpurea* (Cresswell, 1993). It could be the case that competition for prey in larger populations of carnivorous plants will result in lower prey capture rates. We found no evidence of this between our study sites that varied greatly in population sizes (from 12 to >100,000 plants).

Prey capture links the inquiline community and *S. purpurea* pitchers through prey decomposition and nutrient uptake. These communities are a model system for understanding food webs (Baiser et al., 2013; Gray et al., 2012; Sirota et al., 2013; Srivastava et al., 2004). In‐situ, prey composition and quantity likely impact on the inquiline community through influences on resource supply (amount of prey), and substrate quality (type of prey). Freedman et al. (2021) found no impact of prey quantity on *S. purpurea* microbiome diversity, but no one has yet investigated the impact of differences in prey identity on microbiome diversity. Diet is an important determinant of microbiome diversity in animal guts (Reese & Dunn, 2018), with greater microbial diversity resulting from a more diverse diet, so we might expect the same in the *S. purpurea* pitcher microbiome. We demonstrate that both the amount and diversity of prey vary between sites, which may have important consequences for the inquiline community. In addition, the presence of *S. purpurea* in Europe as a non‐native species provides potential for new understanding of food‐web dynamics, because of the absence of some key species in the detritivore community. *Sarracenia purpurea* hosts several obligate species (Addicott, 1974; Błȩdzki & Ellison, 2003; Heard, 1994): the larval stages of pitcher plant flesh fly (*Fletcherimyia fletcheri*), non‐biting midge (*Metriocnemus knabi*), mosquito (*Wyeomyia smithii*), and the pitcher plant mite (*Sarraceniopus gibsoni*). Of these only *S. gibsoni* has been found in *S. purpurea* pitchers in Europe (Goddard et al., 2022). *Wyeomyia smithii* is a keystone species (Gotelli & Ellison, 2006) and so its absence is likely to alter substantially the food‐web dynamics. To fully exploit, this system requires understanding of how the ecology of *S. purpurea* varies (or not) in Europe compared to its native range. We show that, in terms of prey capture at the rank of order, *S. purpurea* functions indistinguishably in its native and non‐native range.

We identified many prey items to species level, which revealed that the most abundant prey species was different at every site. Comparison with *S. purpurea* diet in the species native range at the species level is not possible because no previous studies have identified prey to this taxonomic resolution. These data are influenced by variations in the decomposability of different groups. For example, Diptera tend to retain their characteristics less well, compared to Formicidae and Coleoptera. However, we can still use these data to better understand species‐specific patterns of prey capture. Experimental evidence suggests that ants (Formicidae) are attracted to nectar produced by extrafloral nectaries on the inside of *S. purpurea* hoods (Bennett & Ellison, 2009). This explains the dominance of Formicidae in *S. purpurea* diets. Most of the ant species trapped across all sites were small species from the Myrmicinae subfamily. *Formica picea* lives exclusively on bogs and has a very localized distribution in Britain and Ireland, including Lower Hyde which is where this species was present as trapped prey. One of the common Coleoptera species, *Anaspis maculate*, is a flower visitor and so might also be attracted to this nectar. The other two dominant Coleoptera species, however, are seed or leaf eaters. One, *Ocys harpaloides*, is a ground‐beetle which was found in 9 out of 10 pitchers at Stoke Flat. This suggests local abundance, resulting in haphazard trapping. The other *Sphaeroderma testaceum* is found at three sites, and across multiple pitchers at those sites. This also suggests local abundance and haphazard trapping contributed to the abundance of prey capture for this species.

*Sarracenia purpurea* is a naturalized species in Europe and can be invasive on some bogs. Understanding the ecology and differences in function between the plant's native and non‐native range is essential for understanding and predicting impacts on habitats, and for predicting when and where the species might become problematic. We found no evidence to suggest that the function of *S. purpurea*, in terms of prey capture, differs in its introduced range compared to its native range. But, further research is needed to determine whether the biogeographic patterns present in the native range (e.g. in plant morphology) are also replicated in the non‐native range, and the extent and impact of variability in diet at the rank of species. Here we suggest two principles which should be considered for any future studies: 1. it is likely that in terms of prey capture at the rank of order, *S. purpurea* functions similarly in Europe and North America; 2. in Europe (and presumably also in North America) prey capture varies between populations in important ways—quantity and identity of prey—which may contribute to differences between sites in food‐web dynamics and prey nutrient uptake. These general principles can be harnessed to investigate the *S. purpurea* model system, providing the potential to drive new understanding of controls over carnivorous plant function, food‐web dynamics and microbiome composition.

## AUTHOR CONTRIBUTIONS

**Rebecca Whatmore:** Conceptualization (equal); data curation (supporting); formal analysis (supporting); investigation (lead); methodology (equal); writing – original draft (equal). **Paul J Wood:** Conceptualization (equal); investigation (supporting); methodology (equal); supervision (equal); writing – review and editing (equal). **Ciara M Dwyer:** Formal analysis (equal); visualization (lead); writing – original draft (supporting). **Jonathan Millett:** Conceptualization (equal); data curation (lead); formal analysis (equal); investigation (supporting); methodology (equal); supervision (equal); writing – original draft (equal); writing – review and editing (lead).

## CONFLICT OF INTEREST

The authors declare no conflicts of interest.

### OPEN RESEARCH BADGES

This article has earned an Open Data badge for making publicly available the digitally‐shareable data necessary to reproduce the reported results. The data is available at https://doi.org/10.17028/rd.lboro.20146499.

## Data Availability

Pitcher morphology, prey capture counts, site locations and secondary data are available in the Loughborough University Data Repository (Figshare): Whatmore et al. (2022) https://doi.org/10.17028/rd.lboro.20146499
